# Toward Synthesizing Executable Models in Biology

**DOI:** 10.3389/fbioe.2014.00075

**Published:** 2014-12-19

**Authors:** Jasmin Fisher, Nir Piterman, Rastislav Bodik

**Affiliations:** ^1^Microsoft Research, Cambridge, UK; ^2^Department of Biochemistry, University of Cambridge, Cambridge, UK; ^3^Department of Computer Science, University of Leicester, Leicester, UK; ^4^Electrical Engineering and Computer Science, University of California Berkeley, Berkeley, CA, USA

**Keywords:** executable biology, synthesis, verification, Boolean networks, signaling pathways

## Abstract

Over the last decade, executable models of biological behaviors have repeatedly provided new scientific discoveries, uncovered novel insights, and directed new experimental avenues. These models are computer programs whose execution mechanistically simulates aspects of the cell’s behaviors. If the observed behavior of the program agrees with the observed biological behavior, then the program explains the phenomena. This approach has proven beneficial for gaining new biological insights and directing new experimental avenues. One advantage of this approach is that techniques for analysis of computer programs can be applied to the analysis of executable models. For example, one can confirm that a model agrees with experiments for all possible executions of the model (corresponding to all environmental conditions), even if there are a huge number of executions. Various formal methods have been adapted for this context, for example, model checking or symbolic analysis of state spaces. To avoid manual construction of executable models, one can apply synthesis, a method to produce programs automatically from high-level specifications. In the context of biological modeling, synthesis would correspond to extracting executable models from experimental data. We survey recent results about the usage of the techniques underlying synthesis of computer programs for the inference of biological models from experimental data. We describe synthesis of biological models from curated mutation experiment data, inferring network connectivity models from phosphoproteomic data, and synthesis of Boolean networks from gene expression data. While much work has been done on automated analysis of similar datasets using machine learning and artificial intelligence, using synthesis techniques provides new opportunities such as efficient computation of disambiguating experiments, as well as the ability to produce different kinds of models automatically from biological data.

## Executable Biology

**Figure 1:**
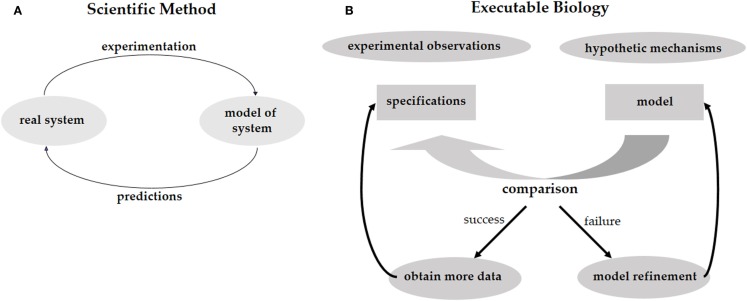
**(A)** The scientific method calls for the elaboration of a predictive model of the system under study. The model should reproduce the existing experimental results and should be predictive regarding future experiments. By performing these experiments we validate the model, or refine it to a better model that captures more facts about the system. **(B)** The same process for executable biology calls for the elaboration of a model in the form of a computer program. The model is compared with specifications obtained from experimental observations. Failure to reproduce the experimental results leads to a refinement of the model. Predictions are used to guide further experimentation.

Investigating phenomena through the scientific method is an iterative process of hypothesis-driven experimentation. We observe the world around us, experiment with it, and, based on the experimental data, come up with hypotheses trying to explain how the systems that we study actually work (Figure 1A). These hypotheses lead to new predictions that then need to be tested in the real world. In biology, working hypotheses are referred to as mechanistic models aiming to provide a mechanistic explanation for observed phenomena. *Executable Biology* is an emerging field focused on the construction of such mechanistic models as executable computer programs. The basic construct of these computer programs (or computational models) is a state-machine, which relates different states to one another by defining how given certain events (e.g., a molecular signal), one state is transformed into another (Fisher and Henzinger, 2007). The components composing such a state-machine represent biological entities, such as cells, proteins, or genes that react to events involving neighboring components by state transformations. These state-machines can then be composed together to form complex computational models representing biological behaviors. As opposed to quantitative mathematical models such as stochastic and dynamic models, computational models are qualitative, as they explain the cause of observed phenomena. A major advantage of qualitative models is that different models can be used to describe the same biological phenomena at different levels of detail (abstraction), and that the different levels can be formally related to one another. For example, models can represent the molecular level, or, at a higher level of abstraction, they may represent the cellular level.

Computational models can be used to test different mechanistic hypotheses. Since the computational model represents a hypothetical mechanism that results in the experimental data, when we execute the model we can formally check whether a possible outcome of the mechanism is consistent with the data. Due to the non-deterministic nature of biological models, it is impossible to exhaustively test that all possible executions of a model conform to the data. Model-checking, on the other hand, is a technique that systematically analyzes all possible outcomes of a computational model without executing them one by one (Clarke et al., 1999). Hence, if model-checking verifies that all possible outcomes of our computational model agree with the experimental data, and that all the experimental observations can be reproduced by the model, then we have a guarantee that the model is realistic and represents a mechanism that fits and explains the data. In case some of the experimental data cannot be reproduced by the model then we know that the hypothesis is wrong. We then need to refine the model until it produces the additional outcomes. Furthermore, if some of outcomes of the computational model disagree with the experimental data then the mechanistic hypothesis represented by the model may be wrong and we would need to revise the model so it will only produce outcomes that are supported by the data. In this case, the refinement of the model will offer new predictions suggesting additional experiments in order to validate the mechanistic hypothesis represented by the model. Executable biology is therefore an interplay between collecting data in experiments and constructing executable models that capture a mechanistic understanding of how a particular system works. By executing these models under different conditions that correspond to the experimental data and comparing the outcomes to the experimental observations, we can identify inconsistencies between hypothetical mechanisms and the actual experimental observations. Similar to the scientific method, this iterative process leads to new hypotheses, which serve to refine the mechanistic model and then need to be validated experimentally (Figure 1B).

Instead of constructing executable mechanistic models manually, one can extract such models automatically from experimental data using a technique called synthesis. Program synthesis is a method used to extract computer programs from their high-level specification. In biology, this concept is extremely appealing, as we would like to avoid the laborious manual process of model construction, which is prone to conscious and unconscious biases and errors, and replace it with an automatic process to synthesize the model directly from the data. Obviously, such a process could yield many different models explaining the same data set, in which case another interesting point would be to identify a way to differentiate between the different models. This could be in the form of an experiment that could either verify or falsify a particular hypothetical model. Hence, automatically synthesizing models of biological programs from experimental data has tremendous advantages over manually constructed biological programs. Usage of synthesis could lead to significant advantages in terms of time and labor to produce models, in terms of our confidence in the inferred features of models, and in terms of the next steps to take to decide between multiple possible models.

## Modeling Methodology

We now present the methodology of executable modeling, describing its steps as a workflow that a biologist might follow when developing and analyzing an executable model. We accompany the explanation with the details of a running example. We exemplify the process through a developmental model of a fragment of the *C. elegans* vulval precursor cells (VPC) system. We will model the lateral signaling mechanism that six adjacent VPC use to collectively determine their fate. The description here follows closely the elaboration of the synthesis process described in (Koksal et al., 2013).

### Choose a suitable abstraction level

Based on the biological question, we choose the level of abstraction, including the biological entities and their possible values (states). The abstraction, coupled with assumptions given by the biologist, defines a space of possible models, and the role of synthesis will be to find models in this candidate space.

For example, we want to develop a model that explains how VPCs coordinate to determine their fate. Specifically, we are interested in (i) identifying which pairwise protein interactions are involved in the coordination; (ii) how the cells gain resilience against “time conflicts,” which arise when two cells happen to signal each other simultaneously; and (iii) how a cell avoids “self-signaling,” which arises when a cell C1 signals its neighbors to take fate 2° – how does C1 ensure that it does not listen to its own signal and incorrectly enter fate 2°? These three modeling questions already dictate which entities and mechanisms must be preserved by the modeling abstraction and which might be abstracted away: the model must have an object per protein to uncover the effect of protein interactions. Similarly, there will be an object per cell, to enable modeling of inter-cell communication. Each such cell model will be composed of multiple proteins that will interact with proteins inside the cell as well as proteins (receptors) on neighboring cells. Finally, to model the effects of different times when signaling happens, our models will, in some fashion, need to model the progress of time.

**Figure 2:**
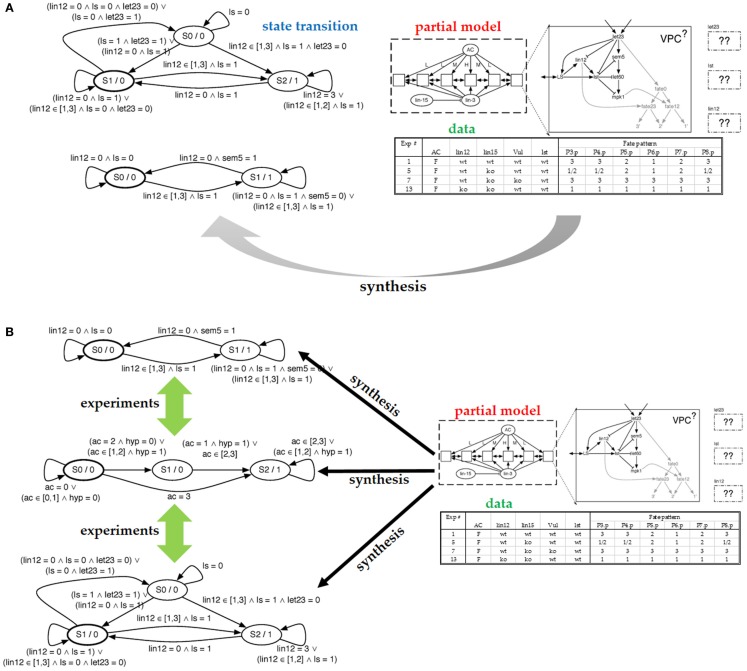
**(A)** Partial model submitted to the synthesizer and the resulting state machine produced by the synthesizer. On the right, we see the structure of a cell with the components that comprise it. We see the configuration of the six cells and the communication allowed between them. Finally, we include some of the experimental data used to specify which models are correct. On the left, we see the resulting state transition diagrams produced for let23 (top) and lst (bottom). **(B)** In the case that the synthesis engine can produce multiple possible models that explain the data, we can ask for experiments that distinguish between the different models. Such experiments are expressed in terms of the experimental setting created for the synthesis effort.

Figure 2A (top right) shows the model of our cell comprises seven proteins and a decision circuit that models how the fates are determined. The figure also shows how six (identical) cells are connected into a system with the anchor cell (AC) and two external proteins communicating the interaction between cells (inter-cellular signaling). To define the model as an executable program, we discretize both time and protein levels. The model will execute in a fixed number of steps (10–15 steps in our setting). Protein concentrations will use a small number of levels (2–5). Discretization levels are set by the biologist. Protein behavior is modeled with a protein response function, which is specific to each protein. The model updates the protein level in each step. The protein response function reads the current level of the protein as well as the levels of incoming proteins, and computes the next state of the protein. The protein response function thus forms a state machine, with one state for each discretized concentration level, and transitions are predicates that test levels of incoming proteins. Figure 2A (left) shows two protein response functions.

To summarize the model semantics, each protein has a state (concentration) to model how the protein concentration evolves over time, which allows for control of when one protein triggers another. Cells are networks of proteins in which all proteins take their step simultaneously (synchronously); there is no need to execute proteins of a cell in arbitrary order, because we are only interested in time-sensitivity between cells. The six cells take steps one after another, controlled by the scheduler that models the different rates of progress of the cells. The role of the scheduler will be explained in the next subsection.

### Collect data and express prior information as a partial model

There are two common methods for narrowing the space of possible models. First, one can state biological “certainties,” such as which proteins are believed to participate in the system under study. Second, one can add the observed biological behaviors that the model needs to reproduce.

Existing biological knowledge will be used to construct an initial model, called a *partial* model, which defines the space of candidate models. We can think of the partial model as a parametric model whose parameters are determined during model synthesis. The partial model may include known biological entities (e.g., proteins), their possible states and interactions. For example, in the model in Figure 2A, the previous biological knowledge included the interactions between the entities and the encapsulation of entities to cells, based on previous data on the behaviors of the entities, the cells, and the VPC system in general. In addition, the structure of the protein response functions was decided based on beliefs regarding the sensing capabilities of the proteins and the assumption that all involved entities are represented in the model. One might also fix the type of interaction between the entities (inducing or inhibiting) and possibly restrict the number of arguments to protein response functions based on the number of active sites of the protein. These prior-belief restrictions define the “structure” of the candidate space and narrow down the search process.

The experimental data are used to test correctness of models in the candidate model space. The experimental data have to be mapped to the model level: the environmental conditions are viewed as the inputs to the model while the experimental observations are viewed as model outputs or intermediate states. In the VPC example, each experiment is a mutation-phenotype pair, where the mutation is the input to the computational model, while the phenotype is the output from executing the model. In more detail, the mutations either knock out a gene or constitutively turn it on. For each mutation, the experimental data records the fate taken by each of the six cells. There are three possible fates, and in each experiment, all cells are identically mutated. Figure 2A (bottom right) shows a portion of the table that maps mutations to fates. Our model will execute by first reading the particular mutation, which changes the behavior of the model. The model is then executed for a predetermined number of time steps, covering the period during which the cells coordinate. When the model terminates, it outputs the six fates.

Another possible example of model output is the time series of a certain combination of entity values. In all cases, the experiments are translated to the same constraint language that is used to express the structural restriction on the models.

Because a model can have multiple executions, we distinguish between allowable behavior and required behavior, using both types of behaviors to decide whether a particular model is correct. In Figure 2A, note that for the second mutation in the mutations-to-fates table, the experiments have observed multiple fates. Presumably, this stochasticity in the cell is due to the loss of “synchronization” between cells caused by the mutation. A correct model will need to be able to reproduce each of the observed fates – they represent the allowable behavior. The required behavior is that each of these alternative fates must be reproducible. That is, there must be an execution of the model that produces one observed fate and another execution that produces the other fate. To endow our model with the ability to reproduce this stochastic behavior, we make the model non-deterministic. We view non-determinism as an abstraction of stochasticity, in that our model will not tell us the probability with which each fate can be reached, only whether it can be reached. The benefit of using non-determinism is that it is not necessary to use randomness to make the model behave stochastically. To make the model non-deterministic, it will suffice to control how the six cells interleave their steps; we call this interleaving a *schedule*. The model includes a scheduler that can non-deterministically select one of the possible schedules.

### Verification of models

The model we have described so far is not specific to synthesis. In fact, one can develop the model entirely manually. It will be desirable to verify this model, which would mean to ensure that for each mutation, the model produces the indicated fates no matter which schedule is selected by the scheduler, and that all alternative fates are produced by some schedule. This verification is performed without explicitly enumerating all schedules, as there are too many. Instead, the model is translated into logical constraints which are supplied to a solver, which in turn is asked to find a schedule that fails to produce the indicated fates. If the solver proves that no such schedule exists, the model has been verified.

### Synthesize and enumerate alternative models

It is usually tedious to manually develop a complete model that verifies against the experiments. To employ synthesis, we ask the synthesizer to complete the partial model into a verifiable model. At the technical level, the synthesis works as a search process. The partial model defines a space of candidate models. Each candidate corresponds to one possible completion of the partial model. Most of the candidate models are typically incorrect (i.e., they disagree with the experimental data), and the synthesizer needs to find a candidate that is correct. As in verification, the search is formulated as solving of a set of constraints. The translation to constraints is performed by a tool (a special compiler) that accepts any partial model and produces logical constraints.

Usually, this will be formed as a type of constraint satisfaction problem, and it is the role of a given solver to search for a solution for it. The translation back and forth between the constraint language and the models and their potential behavior is the main programing task in this endeavor. If the solver manages to find a possible solution then this is a potential model. If the synthesizer fails to find possible models, the prior knowledge and assumptions have to be reconsidered in order to enlarge the search space, for example, by adding additional protein interactions. Having synthesis algorithms produce useful information for such enlargement is an ongoing research topic.

In the VPC case study, the laborious aspect of model development is to write protein response functions that collectively behave as the real cell. Therefore, these response functions will be produced by the synthesizer. These functions will describe how proteins respond to suppression and activation, informally, how long it takes for proteins to become activated. If no such function can be found, we assume that the model is missing an interaction, and the prior knowledge needs to be revised, as done by Fisher et al. (2007). Figure 2A (top right) shows the partial model supplied to the synthesizer; the response functions for three proteins need to be synthesized. Given the table with experimental data and the number of steps to be taken, the synthesizer completes the partial model with protein response functions. Two of the synthesized response functions are shown on the left. This model is correct in that it can be verified as described above.

### Analysis of the space of feasible models

The synthesizer has discovered that multiple models match the data. It is therefore natural to ask whether these models represent alternative match the data. It is therefore natural to ask whether these models represent alternative explanations of how the cells behave. Given how we posed the problem, these models are equivalent, because we have fixed the interaction network and solved for transfer functions. Hence, our models will differ only in their protein responses, which we typically do not consider to be different explanations. To arrive at an alternative explanation, we pose to the synthesizer a different partial model (with a different set of interactions) and ask whether response functions exist for that model. An alternative technique is to give the synthesizer a partial model with a superset of interactions and then read out the synthesized response functions: if a function ignores an incoming protein, then we can remove that incoming edge, arriving at another model. In Figure 2B, we show alternative models synthesized from our partial model.

### Compute additional experiments to conduct

If alternative models exist, it is because we do not possess sufficient experiments to narrow down the candidate space to a single model. To rule out some alternative models, we can ask the synthesizer to compute additional experiments for which the measurable result differs between the alternative models. In the VPC case study, this is done by searching the space of mutations (for which experiments have not yet been performed), looking for a mutation such that at least two alternative models differ in their fate outcomes. Performing this experiment and adding its result to those that guide the synthesis process is guaranteed to rule out some of the models. If no such experiment exists, then, from the point of view of the existing experimental system, it is impossible to distinguish between the alternative models, and additional experimental methods need to be considered in order to facilitate ruling out some of these models.

#### Minimizing the number of experiments

Assume that you want to rerun the experiments, for example, to increase your confidence in the measurements. Do you need to perform all the experiments or is it sufficient to redo a small subset of experiments? Indeed, prior knowledge may make some experiments unnecessary. Alternatively, one or more experiments may collectively make some other experiments superfluous, because no new knowledge useful to model inference is present in the latter set of experiments. This problem is again posed as a search over a sufficient set of experiments that will infer the same set of plausible models as the full set of experiments. In our case study, we were able to reduce the number of experiments from 48 to 4.

## Developing Models with Synthesis

We now aim to generalize some considerations discussed above and present questions that need to be answered in order to apply synthesis effectively in biological domains. We follow the structure of the previous section and revisit the issues that were highlighted there.

### Choose a suitable abstraction level

The first question that needs answering is whether the biological question that we have in mind can be helped by synthesis. At the current level, successful applications of synthesis are restricted to constructing discrete models at a relatively low level of detail^1^. For example, models of continuous evolution of protein networks that match time series expression levels are more suited for other methods. The synthesis techniques we talk about here are based on constraint solving. Such techniques are more appropriate when the values of entities can be represented by discrete values and their changes over time are abstracted to talk about “what happens next,” ignoring the detail of “when exactly” it happens. This dictates a relatively high level of abstraction and questions that relate to, for example, possible interactions, causality, and nature of interaction. The kind of models that can be produced are, for example, Boolean networks (Kauffman, 1969), state transition diagrams (Efroni et al., 2007), and Petri-nets (Bonzanni et al., 2013). Such programs will generally manipulate variables ranging over discrete domains and changing by transition rules that set a next value based on the current value of an entity and those affecting it. The stress on current and next is intentional. It marks a clear difference between the mathematical models that talk about the transformation of values over time and the computational models that we have in mind here.

### Synthesize and enumerate alternative models

One can look for differences or commonalities between all correct models leading to further biological experimentation. For example, one can look on the options synthesized for a certain part of the system. If the set of options is very restricted, this means that the existing knowledge and experiments lead to a good understanding of the structure of this part of the system. If the number of options is very large and potentially contradictory then additional information about this part is missing. In particular, summarizations of the entire space of correct models can give us information on what is impossible (if no model uses such a feature) and what must be true (if all models have such a feature). One of the questions that will need resolution in order to make synthesis more applicable in the biological domain is how to better classify the set of potential models and what kinds of questions can be asked about them, which could also be useful for manual elaboration of models.

### Compute additional experiments to conduct

Similar techniques to those applied to produce the model resulting from synthesis can be used to search the space of experiments. In particular, adding information about the cost of experiments and their feasibility could narrow down the space of possible experiments and suggest that the “easiest” experiments to perform that would still give information valuable in ruling out potential models.

### Over fitting to experiments

One of the issues in parameter estimation techniques is that of over fitting models to noisy and unreliable data, leading to models that are too restrictive. We stress that the approach to tackle over fitting within the context of synthesis must be different. Here, the technique itself is structured so as to determine a space of possible models. It does not make sense to synthesize with only some of the information in hand and then test the resulting models with additional data. Indeed, the step that could lead to over fitting is in the definition of the space of possible models and not in the partition of the space between “correct” and “incorrect” models. First, since the technique can declare that a certain space does not contain “correct” models, the risk of over fitting is somewhat reduced. Over fitting may result in models that are not realistic but can still explain all the observed phenomena. The predictions of such models should lead to the identification of the errors in the definition of the search space. Second, the technique is geared toward the production of multiple models and not the “best fitting model.” The resilience to over fitting should be part of the definition of the space of possible models and the type of correspondence between the models and the experimental results. Removal of some of the experimental results and testing them at a later stage, essentially will lead to either of the two answers that we would have reached in the first place: a narrower space of “correct” solutions or the non-existence of a “correct” solution.

## Additional Examples

We explore a few more examples of the usage of synthesis techniques as described above.

### Synthesizing boolean networks from gene expression experiments

Another example of synthesis is the following application to the extraction of a Boolean network from experimental data (Guziolowski et al., 2013). The existing biological knowledge consists of the connections between the different biological entities along with their directions. That is, the authors assume that they know which proteins interact and, for every interaction, whether the interaction is positive or negative. This information is summarized in the form of a directed and annotated graph (*G* = *V*, *E*), where *V* is a set of nodes, and *E* ⊆ *V* × *V*is the set of edges. The annotation of edges with + and − signs is given separately.

The assumption on the structure of the model is that it is a Boolean network. That is, every biological entity corresponds to a variable that is either on or off (0 or 1), and there are rules that govern the changes in values of these entities according to the values of the entities that have an edge to them. In particular, the function that sets the value of an entity is a Boolean function that includes all the entities that affect the entity we are interested in and where the sign of every interaction is respected. So, an entity that affects positively cannot have an inverse effect with every other possible combination of the other inputs and vice versa. It is well known that such networks stabilize according to these rules. Thus, there are certain states (assignments of values to all the variables) in which updates produce no change.

The experimental framework assumes some inputs *I* ⊆ *V*, which are biological entities that can be affected by experiments, and some outputs *O* ⊆ *V*, which are biological entities that can be measured. Thus, the set of experiments that can be done on this network are to set the values of the inputs (by mutation or other intervention) and to measure the values of the outputs (e.g., phosphorylation values). Outputs are discretized to a number of levels that correspond to noise level in the measurements. In this particular case, the output is discretized to 100 levels. It is assumed that the output values that are associated with a certain input correspond to the stability point of the network when the inputs are set to the experimental value.

The utility of the network is measured by the sum of the square distance of the measured outputs from the output of the network. For a specific experiment *e* and specific output *o* ∈ *O*, we write θ*_e,o_* as the value of this output in this experiment. Similarly, given a candidate Boolean network, the stability value of a certain output under the same experiment is denoted ρ*_e,o_*. The distance of a specific experiment is *d_e_* = 1/*m*∑*_o_*(ρ*_e,o_* − θ*_e,o_*)^2^, where *m* = |*O*|. That is, the average of the square of the distance from the network prediction and the actual experimental results over all outputs. The overall distance of the network is the average of the distances overall experiments, that is *d* = 1/*n*∑*_e_d_e_*, where *n* is the number of available experiments. The network utility is to minimize the distance of the network from experiments and at the same time minimize the size of the network (as measured by the size of the functions that govern changes in variable values).

The authors of this research then pose this question as a search question. Find the network that best matches the experimental data with the minimal size. The initial search yields 16 models that result from allowing two possible functions for four entities (2^4^ = 16 – not very surprising as searching for a minimum often does). But relaxing the requirements to within a distance of 10% from the possible minimum to take noise into account produces about 10,000 possible networks.

The main strength of this analysis technique is that it is now possible to analyze all these 10,000 networks simultaneously and derive conclusions from their commonalities. For example, for some entities the same functions occurred in *all* the models. Functions that do not appear in even a single model are overruled. And some entities had a very small number of possible functions. In addition, the analysis can extract whether it is possible to perform experiments within the given experimental framework that will distinguish between models. That is, design an experiment over the given inputs so that the two different networks will have different values for the given outputs, and would thus be measurable by a given experiment. Every two models that cannot be distinguished by the experiments are considered equivalent from the point of view of the experimental setting and they found that there are 91 such classes of models. They proceed to propose experiments that will overrule some of these model classes.

The work of Sharan and Karp (2013) uses different underlying techniques for searching; however, their definition of the problem is quite similar to the setting above. As in the previous work, they search for Boolean networks. Their assumptions regarding the knowledge about possible interactions is weaker, in that they do not assume that they know the directions of interaction and also accept if interactions do not have an inhibition/activation annotation. As a result, they allow more general functions for the next state function of individual entities in the Boolean network. They use the same measurement for the distance between the experimental results and the network behavior. Finally, the underlying solving techniques are through integer linear programing (ILP) and not constraint (or Boolean) solving as we mostly do here.

A similar application of synthesis to extract Boolean networks is in (Dunn et al., 2014). The authors again search for a Boolean network that matches a given experimental setting. Here, the assumptions are somewhat different, leading to some different choices. The relevant biological data correspond to possible connections between the entities but this time without directionality and without the label of activation/inhibition. Accordingly, it is the role of the synthesis engine to find the exact connections as well as the way that entities affect each other. The update functions for the entities are restricted to a small set of possible functions. The last choice significantly narrows the search space. The treatment of the experimental data is also different. Here, the authors do not assume the existence of inputs but rather search for an execution of the network from a given initial state corresponding to the experimental setting to a final state corresponding to the measurements. This time the experimental data are made Boolean by the authors and the matching between an experimental measurement and the state of the Boolean network has to be exact. As before, the authors summarize all possible models in the space that match the experimental data. They draw conclusions regarding common features of all these models and experimentally verify some of their predictions.

## Future Prospects and Open Problems

In all the cases discussed previously, executions of models were considered bounded and of a certain length. It would be interesting to lift this restriction and relieve the modeler from the need to make this decision. Techniques that support such synthesis efforts are in general more complicated, and it would be very interesting to see them adapted for the biological context (Vardi, 2008; Kupferman, 2012).

The level of abstraction we discussed above is very convenient for synthesis. We assume that genes have discrete levels of expression. It would be very interesting to devise techniques that produce models at various levels of abstraction, for example, accompany the transfer functions supplied above by molecular interaction models that could give rise to the same behavior.

In the detailed case study presented in an earlier section, the model reproduced stochastic behavior with non-determinism based on Boolean logic: a particular fate could either happen or not happen. Logical modeling was sufficient in that case study, because both the inputs to the model (mutations) and the outputs (fates) were discrete values. In modeling situations where the data are quantitative and noisy, the modeling may need to prevent discretization and may require stochastic reasoning with probabilistic distributions, requiring that we change our underlying reasoning engine from a logical one to a probabilistic one [see e.g., Fränzle et al. (2008)]. Genomic high-throughput data falls into this category. Much more work is needed to make this modeling transition.

When one considers synthesis, the most important part of the synthesizer is the compiler that translates a partial model into constraints. The construction of the compiler can be laborious, especially if the model has advanced semantics, as it did in our detailed case study in an earlier section. To simplify the process of creating the compiler, it may be possible to rely on the recently developed symbolic virtual machine (Torlak and Bodik, 2014), which allows one to define the modeling language in a simple way, by writing a so-called interpreter. The symbolic virtual machine produces the compiler automatically from the interpreter. As we have suggested, making such a tool easier for domain experts to use is required and far from accomplished. The efforts described above rely on extensive collaborations between biologists and computer scientists. Gaining more experience in synthesis at a level that will allow the creation of custom level tools that can be used for general synthesis projects, rather than being custom made for a certain synthesis effort is a very ambitious goal. Making such tools usable by domain experts (biologists) is a further challenge. This also implies that at this stage, potential users of the technique cannot rely on existing tools and must invest in the development of synthesis engines for their own needs.

Further case studies should also consider the size boundary applied in the case studies described earlier. The current limit of such techniques is applications to systems with a few tens of proteins (working on standard desktop computers). Scaling the techniques applied above to hundreds of proteins is a major challenge that will require improvements to the underlying solvers as well as finding more efficient ways to encode the specific synthesis questions arising from biology in better ways.

## Concluding Remarks

In recent years, we have seen an increase in the usage of executable biology for modeling various biological systems and phenomena. Advantages, such as the ability to automatically check whether a model adheres to requirements arising from biological data and to answer further queries about the model, make this set of techniques more applicable. In computer science, the research on verification of models has led to work on automatic synthesis of models from their high-level descriptions. Here, we give a short survey of this technique and how it can be used for biological modeling. We summarize some of the main instances where synthesis has been applied to the production biological models and how this extra power gives further insights into the model in question. Finally, we also discuss some of the future developments needed in order to make this technique more applicable for biological research.

## Conflict of Interest Statement

The authors declare that the research was conducted in the absence of any commercial or financial relationships that could be construed as a potential conflict of interest.

## Footnotes

^1^We consider work for parameter estimation of networks of differential equations as belonging to a different class of applications. Indeed, the technique it relies on is completely different to those used here. For a survey of available techniques for parameter estimation we refer the reader to Jianyong et al. (2012).
